# High-resolution free-breathing automated quantitative myocardial perfusion by cardiovascular magnetic resonance for the detection of functionally significant coronary artery disease

**DOI:** 10.1093/ehjci/jeae084

**Published:** 2024-03-25

**Authors:** R Crawley, K P Kunze, X Milidonis, J Highton, S McElroy, S M Frey, D Hoefler, C Karamanli, N C K Wong, S J Backhaus, E Alskaf, R Neji, C M Scannell, S Plein, A Chiribiri

**Affiliations:** School of Biomedical Engineering & Imaging Sciences, King’s College London, St Thomas’ Hospital, Westminster Bridge Road, London, SE1 7EH, UK; School of Biomedical Engineering & Imaging Sciences, King’s College London, St Thomas’ Hospital, Westminster Bridge Road, London, SE1 7EH, UK; Magnetic Resonance Research Collaborations, Siemens Healthcare Limited, Camberley, UK; School of Biomedical Engineering & Imaging Sciences, King’s College London, St Thomas’ Hospital, Westminster Bridge Road, London, SE1 7EH, UK; DeepCamera MRG, CYENS Centre of Excellence, Nicosia, Cyprus; School of Biomedical Engineering & Imaging Sciences, King’s College London, St Thomas’ Hospital, Westminster Bridge Road, London, SE1 7EH, UK; Aival, London, UK; School of Biomedical Engineering & Imaging Sciences, King’s College London, St Thomas’ Hospital, Westminster Bridge Road, London, SE1 7EH, UK; Magnetic Resonance Research Collaborations, Siemens Healthcare Limited, Camberley, UK; Department of Cardiology, University Hospital Basel, Basel, Switzerland; Department of Radiotherapy, University of Erlangen, Erlangen, Germany; School of Biomedical Engineering & Imaging Sciences, King’s College London, St Thomas’ Hospital, Westminster Bridge Road, London, SE1 7EH, UK; School of Biomedical Engineering & Imaging Sciences, King’s College London, St Thomas’ Hospital, Westminster Bridge Road, London, SE1 7EH, UK; Department of Cardiology, Campus Kerckhoff of the Justus-Liebig-University Giessen, Kerckhoff-Clinic, Bad Nauheim, Germany; School of Biomedical Engineering & Imaging Sciences, King’s College London, St Thomas’ Hospital, Westminster Bridge Road, London, SE1 7EH, UK; School of Biomedical Engineering & Imaging Sciences, King’s College London, St Thomas’ Hospital, Westminster Bridge Road, London, SE1 7EH, UK; School of Biomedical Engineering & Imaging Sciences, King’s College London, St Thomas’ Hospital, Westminster Bridge Road, London, SE1 7EH, UK; Department of Biomedical Engineering, Eindhoven University of Technology, Eindhoven, the Netherlands; School of Biomedical Engineering & Imaging Sciences, King’s College London, St Thomas’ Hospital, Westminster Bridge Road, London, SE1 7EH, UK; Leeds Institute of Cardiovascular and Metabolic Medicine, University of Leeds, Leeds, UK; School of Biomedical Engineering & Imaging Sciences, King’s College London, St Thomas’ Hospital, Westminster Bridge Road, London, SE1 7EH, UK

**Keywords:** cardiovascular magnetic resonance, CMR, myocardial perfusion, coronary artery disease, quantitative perfusion, myocardial blood flow

## Abstract

**Aims:**

Current assessment of myocardial ischaemia from stress perfusion cardiovascular magnetic resonance (SP-CMR) largely relies on visual interpretation. This study investigated the use of high-resolution free-breathing SP-CMR with automated quantitative mapping in the diagnosis of coronary artery disease (CAD). Diagnostic performance was evaluated against invasive coronary angiography (ICA) with fractional flow reserve (FFR) measurement.

**Methods and results:**

Seven hundred and three patients were recruited for SP-CMR using the research sequence at 3 Tesla. Of those receiving ICA within 6 months, 80 patients had either FFR measurement or identification of a chronic total occlusion (CTO) with inducible perfusion defects seen on SP-CMR. Myocardial blood flow (MBF) maps were automatically generated in-line on the scanner following image acquisition at hyperaemic stress and rest, allowing myocardial perfusion reserve (MPR) calculation. Seventy-five coronary vessels assessed by FFR and 28 vessels with CTO were evaluated at both segmental and coronary territory level. Coronary territory stress MBF and MPR were reduced in FFR-positive (≤0.80) regions [median stress MBF: 1.74 (0.90–2.17) mL/min/g; MPR: 1.67 (1.10–1.89)] compared with FFR-negative regions [stress MBF: 2.50 (2.15–2.95) mL/min/g; MPR 2.35 (2.06–2.54) *P* < 0.001 for both]. Stress MBF ≤ 1.94 mL/min/g and MPR ≤ 1.97 accurately detected FFR-positive CAD on a per-vessel basis (area under the curve: 0.85 and 0.96, respectively; *P* < 0.001 for both).

**Conclusion:**

A novel scanner-integrated high-resolution free-breathing SP-CMR sequence with automated in-line perfusion mapping is presented which accurately detects functionally significant CAD.

## Graphical Abstract

**Graphical Abstract:**
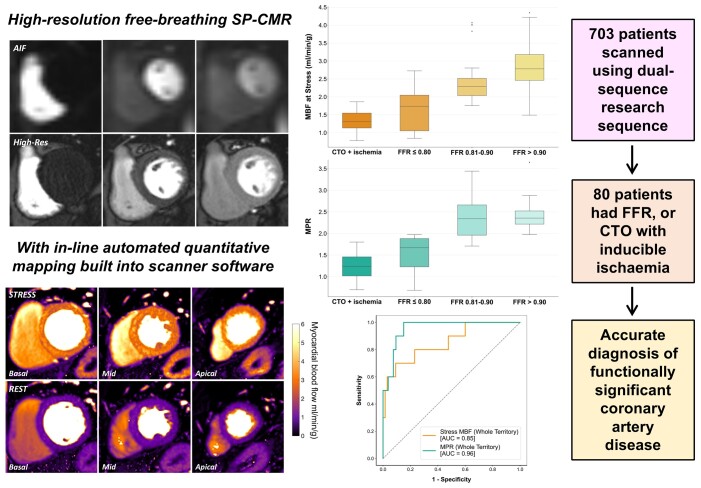
High-resolution free-breathing stress perfusion cardiovascular magnetic resonance (SP-CMR) with in-line automated quantitative mapping is accurate in the diagnosis of functionally significant coronary artery disease. Diagnostic performance was compared with invasive fractional flow reserve (FFR) with myocardial blood flow (MBF) at hyperaemic stress and myocardial perfusion reserve (MPR) reduced in patients with inducible ischaemia. CTO, chronic total occlusion; AUC, area under the curve.

**See the editorial comment for this article ‘Quantitative myocardial perfusion and the power of numbers’, by J. Schwitter, https://doi.org/10.1093/ehjci/jeae108.**

## Introduction

Stress perfusion cardiovascular magnetic resonance imaging (SP-CMR) is recommended as a first-line non-invasive investigation for patients deemed to have intermediate- to high-risk of coronary artery disease (CAD).^1,2^ For diagnosis of CAD, risk stratification and as a guide to revascularization, SP-CMR is comparable/superior to other first-line non-invasive investigations.^3–7^ Whilst fractional flow reserve (FFR) measurement from invasive coronary angiography (ICA) remains the clinical reference standard for the assessment of functionally significant CAD, SP-CMR-guided management has been shown to be non-inferior to FFR-guided treatment strategies.^8,9^ Improvements in the spatial resolution of SP-CMR have demonstrated an increase in both diagnostic accuracy and reporter confidence.^10,11^ However, the interpretation of SP-CMR in current practice still relies on visual evaluation, requiring a high level of reporter experience, training and confidence to draw accurate conclusions.^12^

Quantitative estimation of myocardial blood flow (MBF) via SP-CMR is accurate in the diagnosis of CAD and superior to visual assessment in identifying complex multi-vessel disease.^13–15^ SP-CMR quantification methods have been validated against both positron-emission tomography and FFR.^16–19^ The methods used in these studies, though, relied on extensive manual post-processing to establish MBF values, which were both time- and labour-intensive.

Fully automated methods for quantification of MBF have been developed to streamline post-processing and make quantification in SP-CMR more applicable to the clinical workflow.^20,21^ Implementation of these pipelines has required the development of several techniques, including respiratory motion-compensation and gadolinium-concentration estimation.^22–24^ However, existing automated pipelines often require additional post-processing capabilities, limiting their deployment to specialist research centres.

This study demonstrates the use of a novel high-resolution free-breathing perfusion sequence with automated in-line quantitative MBF mapping in patients under investigation for stable chest pain. The free-breathing sequence is deployed directly within the scanner operating software and is capable of reconstructing high-resolution pixel-wise quantitative maps visible on the scanner console within 2–3 min, allowing real-time interpretation of findings. Earlier iterations and individual components of this framework have been described previously.^25–27^

The aim of this study was to establish the diagnostic accuracy of this high-resolution automated quantitative perfusion mapping pipeline to identify functionally significant CAD in clinical patients. The accuracy of the sequence was evaluated by comparison of the automatically generated MBF values with corresponding ICA findings and FFR measurements.

## Methods

### Study population

Patients aged 18–90 were prospectively recruited between January 2022 and May 2023 at St Thomas’ Hospital, London, UK. The patients were referred for investigation of CAD by SP-CMR based on the reported symptoms. All provided written consent as part of a large cohort observational study, with ethical approval provided by the North of Scotland Research Ethics Committee (ref. 15/NS/0030). All participants underwent high-resolution SP-CMR with in-line quantitative mapping visible to the reporting clinicians. Participants were included for analysis if they underwent ICA (performed by the patient’s clinical team) within 6 months of their SP-CMR scan (before or after); patients were excluded in the event of change in anti-anginal medication, myocardial infarction, or coronary revascularization within this timeframe. Patients receiving clinically indicated FFR evaluation in one or more coronary arteries were included in the ‘FFR’ study subgroup. Patients with an occlusion within the proximal-to-mid portions of a major epicardial coronary artery, along with the visual identification of an inducible perfusion defect in the same territory (exceeding the boundaries of any infarction scarring seen on late gadolinium-enhanced imaging), were included within the ‘chronic total occlusion (CTO)’ study subgroup. Patients were excluded from this subgroup if there was evidence of transmural/near-transmural infarction scarring within the territory.

### Perfusion image acquisition

All patients underwent SP-CMR on a single 3T scanner (MAGNETOM Vida, Siemens Healthineers AG, Erlangen, Germany) with an 18-channel flexible phased array coil and 72-channel spine coil array. Subjects were asked to abstain from nicotine and caffeinated products for 24 h prior to the SP-CMR scan. Perfusion imaging consisted of 60–70 dynamic acquisitions using a saturation recovery fast gradient echo research sequence in free breathing. A bolus of 0.075 mmol/kg gadobutrol (Gadovist, Bayer AG, Leverkusen, Germany) was administered at 4 mL/s with 20 mL saline flush during each acquisition. For high-resolution perfusion acquisitions, three short-axis slices of the left ventricle (LV) were imaged during each R–R interval using a temporally varying k-space sampling pattern with a total acceleration factor of 5.^27^ Typical imaging parameters were: 1.4 × 1.4 mm^2^ acquired in-plane resolution, 8-mm slice thickness, field of view (FOV) 380 × 300 mm^2^, echo time (TE) 1.17 ms / repetition time (TR) 2.62 ms, time from saturation to k-space centre 93 ms, slice acquisition time 113 ms, flip angle 15°. For acquisition of the arterial input function (AIF), a dual sequence approach was employed—low-resolution AIF images were acquired at the basal level of the short axis at the start of each R–R interval.^28^ Typical imaging parameters for the AIF acquisition were: 5.9 × 5.9 mm^2^ in-plane resolution, 10  mm slice thickness, FOV 380 × 300 mm^2^, TE/TR 0.68/1.18 ms, linear k-space ordering, time from saturation to k-space centre 27  ms, slice acquisition time 46  ms, flip angle 8°. The first dynamics for both AIF and high-resolution series were acquired as proton density (PD) weighted images without saturation preparation and reduced flip angle (5°).

Imaging was performed both during hyperaemia and at rest (≥10 min following stress image acquisition). Hyperaemic vasodilatory stress was achieved using either adenosine or regadenoson, directed by the supervising physician in keeping with local safety protocols. Adenosine was administered as a continuous infusion with the dose (140/175/210 μg/kg/min) titrated to both patient heart rate and symptomatic response. Imaging was performed at 3–6  min after commencement of the infusion. Regadenoson was administered as a 400 μg bolus with 10 mL saline flush, with imaging typically conducted at 2 min after administration.

### Image reconstruction and perfusion mapping

**Figure 1:**
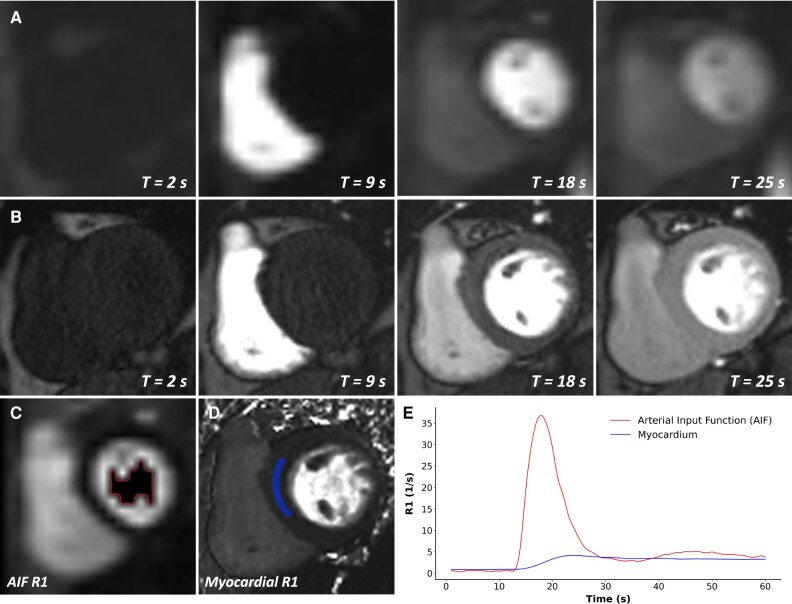
Contrast transit over time during sequence acquisition in a patient with normal MBF. Row (*A*) represents AIF slice acquisition over time and row (*B*) demonstrates the basal myocardial slice at the same time points. (*C*) Automated segmentation of the AIF longitudinal relaxation rate (R1) map series used as a region of interest (ROI) for assessment of the AIF. (*D*) Basal myocardial R1 series with an ROI placed in the interventricular septum. (*E*) Time–R1 curves of both the automated AIF ROI and manual ROI seen in the R1 series.

The entire pipeline for image reconstruction and perfusion map generation was implemented in-line, within the scanner software as a research tool and without the use of any external hardware. Reconstruction of high-resolution perfusion images featured an iterative framework with motion compensation integrated into the temporal regularization as described previously.^24,25,27^ For perfusion mapping, a trained convolutional neural network was used to identify the LV and deploy a bounding box to crop both the high-resolution myocardial series and the retrospectively motion-corrected AIF series. For all time frames within both of these series, image signal inside the bounding box was converted to gadolinium concentration via Bloch equation signal modelling, with PD images used for normalization.^23,29^ Automated segmentation of the LV blood pool allowed AIF–time curve sampling from the gadolinium-converted AIF series (*Figure 1*). Finally, a Fermi-constrained deconvolution was applied to the first-pass section of each pixel time curve of the myocardial series, allowing pixel-wise estimation of MBF.^30^ All images and maps were available to view on the scanner console—average image reconstruction times were 1–2 min, with processing times of the perfusion mapping pipeline adding approximately 30 s–1 min for all the three slices.

### Image analysis

All scans were reviewed by two accredited physicians with >3 years cardiovascular magnetic resonance (CMR) experience to establish the presence of inducible perfusion defects from visual analysis of all perfusion and late gadolinium enhancement (LGE) images. Reviewers were asked whether there was evidence of inducible regional hypo-perfusion during first-pass transit of gadolinium through the myocardial slices. Where there was disagreement, a third experienced reporter opinion was sought. Assessment of heart rate response to vasodilator (considered adequate if ≥10 beats per minute) and the presence of splenic switch-off sign (in those administered with adenosine) were used to evaluate the hyperaemic stress response in all cases considered for analysis.^31,32^ Patients were included in the analysis if one or both of these criteria were fulfilled; if neither was seen, patients were excluded.

**Figure 2:**
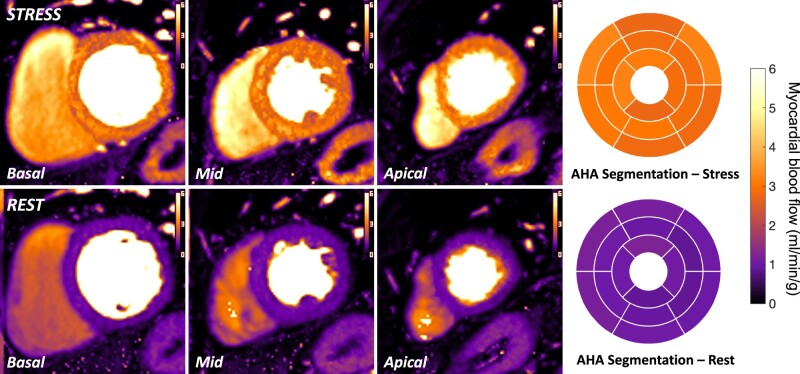
Example of perfusion maps in a patient with no evidence of CAD [FFR in the left anterior descending (LAD) artery 0.90, other vessels not assessed]. Global stress MBF 2.97 mL/min/g, global rest MBF 1.03 mL/min/g. The corresponding AHA segmentations for both stress and rest MBF maps are demonstrated.

Perfusion maps were analysed offline using MATLAB (Mathworks Inc, Natick, MA, USA). Endocardial and epicardial contours were delineated and right ventricular insertion points identified to segment the myocardium based on the American Heart Association (AHA) 17-segment myocardial model (the apical cap was not imaged) (*Figure 2*). The analysis tool then registered 600 sampling points within each slice (60 circumferential points within 10 transmural layers). Mean pixel-based MBF values were calculated if the sampling points bridged multiple pixels. Segmental MBF was calculated as the mean of the MBF values of the sampling points within each AHA segment. Myocardial perfusion reserve (MPR) for each AHA segment was calculated as the ratio of mean segmental MBF at hyperaemia (stress MBF) and mean segmental MBF during resting conditions (rest MBF). An estimation of total coronary territorial perfusion was calculated as the mean of the corresponding segmental MBF and MPR values in AHA segments traditionally associated with each coronary territory.^33^ Segmental and territorial perfusion values were extracted and organized using Python code. To allow comparison with previous studies, the mean of the two lowest scoring stress MBF values from AHA segments within each coronary territory were also considered (‘lowest 2 segment’ method).^16,17^ The corresponding MPR was calculated using the mean segmental MPR values from these same segments at rest.

### Statistical analysis

Analysis was conducted using SPSS Statistics version 29 (IBM, Armonk, NY, USA). Shapiro–Wilk testing demonstrated that segmental and territorial stress and rest MBF values were not normally distributed amongst the population. As such, continuous variables are represented as median (inter-quartile range), whilst categorical variables are represented as frequencies and corresponding percentage. Correlation of non-parametric continuous variables was evaluated by the Spearman correlation coefficient. The comparison of statistical significance among multiple categorical groups was conducted using a Mann–Whitney *U* test for two categories, and Kruskal–Wallis testing for more than two categories. Receiver operating characteristic (ROC) analysis was used to assess the diagnostic accuracy of quantitative datasets. Calculation of both sensitivity and specificity was established using a Youden index to identify optimal cut-offs for both stress MBF and MPR. Area under the curve (AUC) values is represented alongside asymptotic 95% confidence intervals. McNemar’s test was used to compare sensitivity and specificity of different analysis methods. Significance was assessed via two-sided testing and a *P*-value of <0.05 was considered statistically significant. All graphs and plots were generated directly from the data via Python code.

## Results

Seven hundred and three patients receiving SP-CMR were recruited in total. Two hundred and eight recruited patients had invasive angiography within 6 months of their clinical SP-CMR investigations. Of these, 60 patients had pressure wire-guided FFR assessment performed, and another 20 patients were identified as having an inducible perfusion defect in the same coronary territory as an epicardial CTO. Only these patients were selected for analysis. The baseline characteristics for each group are shown in *Table 1*. Ten FFR measurements were assessed as abnormal and functionally significant, as defined by FFR value ≤0.80. All other FFR measurements were >0.80, and therefore deemed to be not functionally significant, irrespective of the presence of visually identified lesions on the ICA.

**Table 1:** Baseline characteristics at the time of CMR scan

	FFR group (*n =* 60)	CTO group (*n =* 20)
Age	61.5 (56.0–71.0)	62.0 (55.5–70.5)
Sex		
Male	31 (51.7)	13 (65.0)
Stress agent		
Adenosine	56 (93.3)	19 (95.0)
Left ventricular ejection fraction	59.0 (55.0–64.0)	47.0 (39.5–58.0)
Cardiac rhythm		
Sinus rhythm	58 (96.7)	20 (100.0)
Atrial fibrillation	2 (3.3)	0 (0.0)
History of CAD	24 (40.0)	14 (70.0)
Previous coronary intervention		
None	48 (80.0)	11 (55.0)
PCI	11 (18.3)	6 (30.0)
CABG	1 (1.7)	3 (15.0)
Diabetes mellitus	19 (31.7)	13 (65.0)
Hypercholesterolaemia	40 (66.7)	11 (55.0)
Hypertension	34 (56.7)	16 (80.0)
Smoking history		
None	36 (60.0)	8 (40.0)
Ex-smoker	20 (33.3)	12 (60.0)
Current smoker	4 (6.7)	0 (0.0)
Presence of LGE (any myocardial segment)	22 (36.7)	18 (90.0)
Visual interpretation of perfusion defect		
Regional hypo-perfusion	10 (16.7)	20 (100.0)
No regional hypo-perfusion	50 (83.3)^a^	0 (0.0)

Continuous variables displayed as median (inter-quartile range); discrete variables displayed as frequency (group %).

PCI, percutaneous coronary intervention; CABG, coronary artery bypass grafting.

^a^Four patients within this group had a poor response to vasodilator and were not included in analysis.

Four patients who received both invasive FFR and SP-CMR were excluded from the quantitative analysis due to poor hyperaemic response to the vasodilator during the SP-CMR. Reasons for poor hyperaemic response included consumption of caffeine or nicotine products prior to the scan, or ineffectual/under-estimation of vasodilator administration. Stress perfusion values were analysed without MPR calculation in nine patients—the reasons for this included rest perfusion imaging not being performed, the use of regadenoson as vasodilatory agent, and the identification of significant artefact within the rest perfusion map.

Qualitative visual assessment of the myocardial perfusion series (both with and without motion compensation) was performed for all the included patients. Ten patients were adjudged to have regional hypo-perfusion defects. Seven of these were found to have one or more FFR-positive lesions via ICA. The remaining three patients were found to have FFR >0.80 in the presumed ischaemic coronary territories. Additionally, one patient with no evidence of regional hypo-perfusion on visual assessment was found to have a single positive coronary territory on FFR assessment.

### Relationship between FFR and territorial CMR quantitative perfusion values

**Figure 3:**
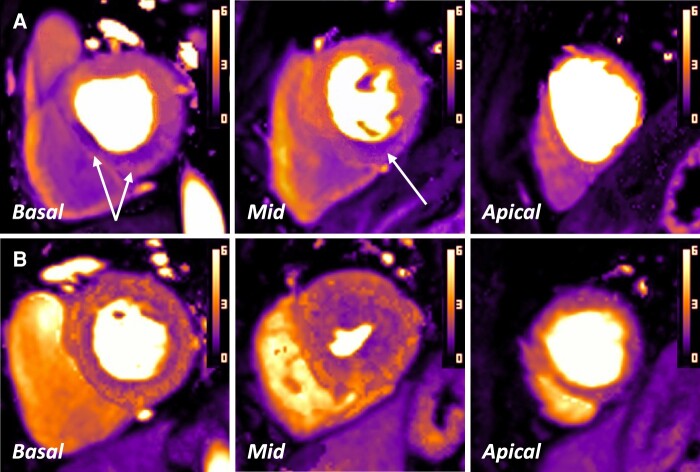
Automated quantitative perfusion maps from two different patients at hyperaemic stress with inducible perfusion defects identified. (*A*) Defect within the inferior/inferoseptal segments, highlighted by white arrows. LGE imaging was normal. FFR in the right coronary artery (RCA) = 0.69. RCA territory stress MBF = 1.50 mL/min/g; MPR = 1.58. (*B*) Near-circumferential epicardial-to-endocardial gradient in the basal and mid-slices, suggestive of microvascular dysfunction. Corresponding FFR in the LAD artery = 0.87; circumflex artery (LCx) = 0.96. LAD territory stress MBF = 2.29 mL/min/g; MPR = 2.73. LCx territory stress MBF = 2.06 mL/min/g; MPR = 2.53.

In total, 75 global coronary artery FFR measurements were matched to the corresponding coronary territorial MBF values obtained at peak hyperaemic stress. Where rest imaging was included in analysis, a total of 63 territorial FFR-to-MPR values were obtained. Examples of automated maps from these patients are shown in *Figure 3*. In two patients, the apical AHA segments were not included in calculating the mean territorial perfusion values due to artefact within the extrapolated maps at peak stress.

**Figure 4:**
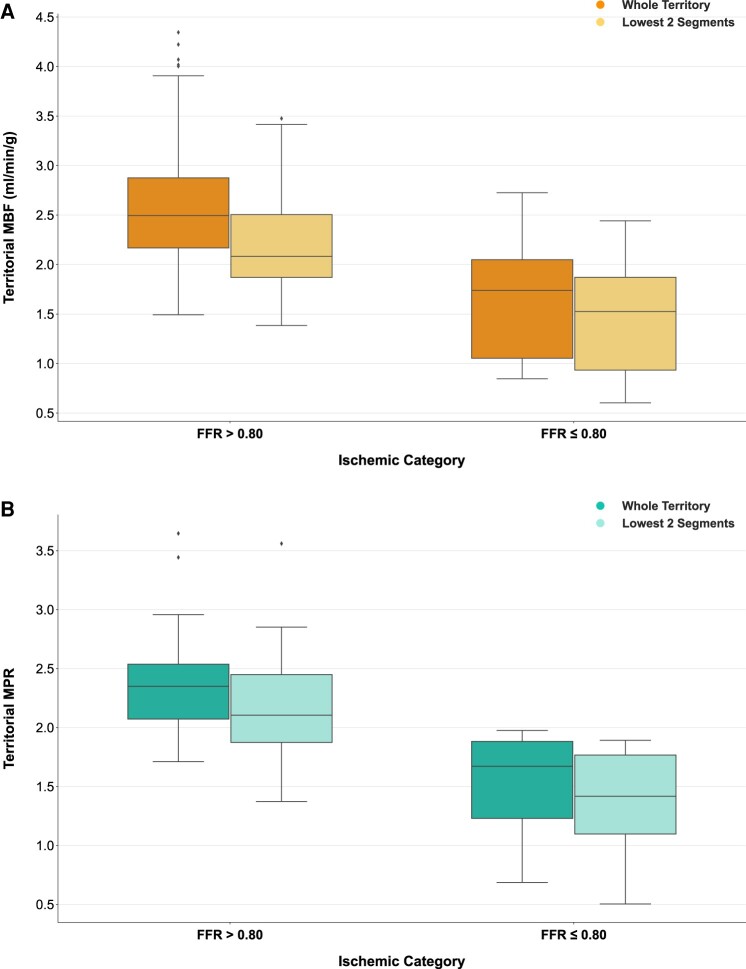
Comparison of perfusion values from whole territory and lowest two AHA segments analysis methods: (*A*) stress MBF and (*B*) MPR. Both stress MBF and MPR were lower in FFR-positive territories, regardless of the method used for assessment.

**Figure 5:**
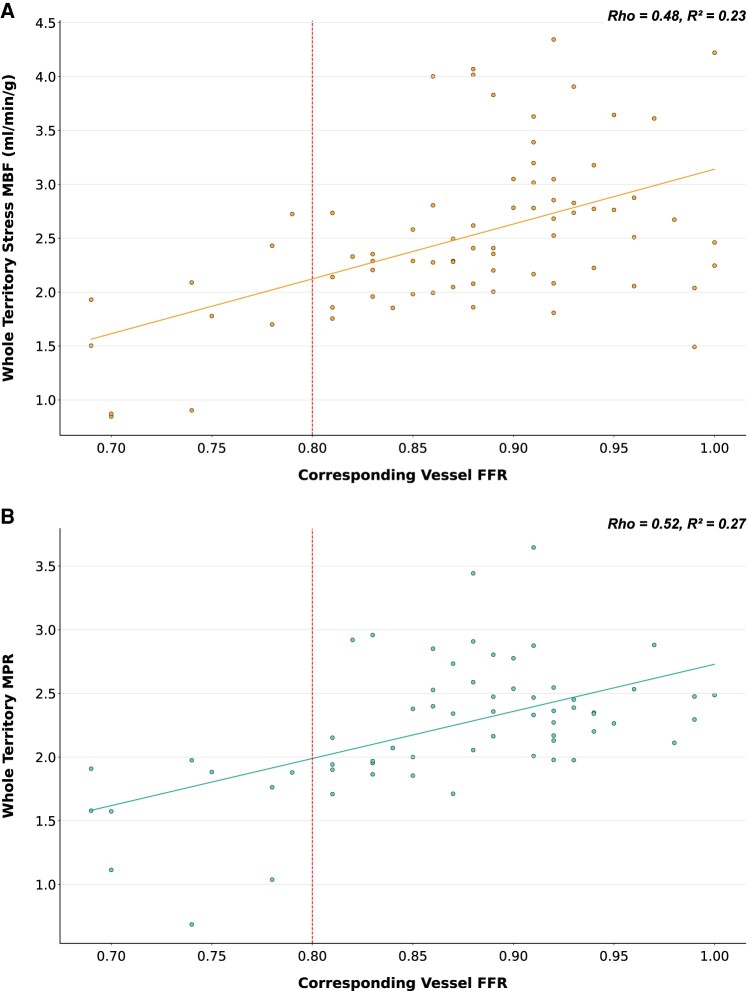
The relationship between FFR and territorial stress MBF (*A*) and MPR (*B*) in all vessels. A positive correlation is seen for both (Rho and *R*^2^ values as shown, *P* < 0.001 for both stress MBF and MPR). The dashed lines (at FFR = 0.80) represent the boundary between functionally normal and abnormal FFR values.

In vessels with FFR measurement ≤0.80, both territorial median stress MBF and MPR were reduced compared to those territories supplied by vessels where FFR >0.80 (median stress MBF: 1.74 (0.90–2.17) mL/min/g, FFR ≤ 0.80, vs. 2.50 (2.15–2.95) mL/min/g, FFR > 0.80, *P* < 0.001; median MPR: 1.67 (1.10–1.89), FFR ≤ 0.80, vs. 2.35 (2.06–2.54), FFR > 0.80, *P* < 0.001) (*Figure 4*). There was no significant difference in rest MBF measurements between the two categories [median rest MBF: 1.04 (0.90–1.40) mL/min/g, FFR ≤ 0.80, vs. 1.10 (0.90–1.29) mL/min/g, FFR > 0.80, *P* = 0.821]. There was a clear linear correlation between FFR and both stress MBF and MPR values (territorial stress MBF: Rho = 0.48, *P* < 0.001; territorial MPR: Rho = 0.52, *P* < 0.001) (*Figure 5*). No significant correlation was seen between rest MBF and FFR (territorial rest MBF Rho = 0.07, *P* = 0.604).

### Assessment of the lowest two segments in each territory vs. FFR

The analysis was repeated by identifying the mean stress MBF of the lowest two AHA segments from each territory and correlating the mean perfusion values from these segments with the corresponding FFR. Again, both median stress MBF and MPR were significantly reduced in coronary territories with FFR ≤ 0.80 compared with the perfusion values from territories with normal FFR measurements [median stress MBF: 1.52 (0.79–1.90) mL/min/g, FFR ≤ 0.80, vs. 2.08 (1.87–2.52) mL/min/g, FFR > 0.80, *P* < 0.001; median MPR: 1.42 (0.99–1.78), FFR ≤ 0.80, vs. 2.11 (1.86–2.45), FFR > 0.80, *P* < 0.001] (*Figure 4*). Similarly to whole territory analysis, there was no significant difference between rest MBF values when assessing the lowest two segments [median rest MBF: 1.02 (0.91–1.37) mL/min/g, FFR ≤ 0.80, vs. 1.03 (0.83–1.21) mL/min/g, FFR > 0.80, *P* = 0.631]. There was a very similar linear correlation between FFR, and both stress MBF and MPR values using the lowest two-segment method (territorial stress MBF: Rho = 0.49, *P* < 0.001; territorial MPR: Rho = 0.51, *P* < 0.001).

### ROC analysis

**Figure 6:**
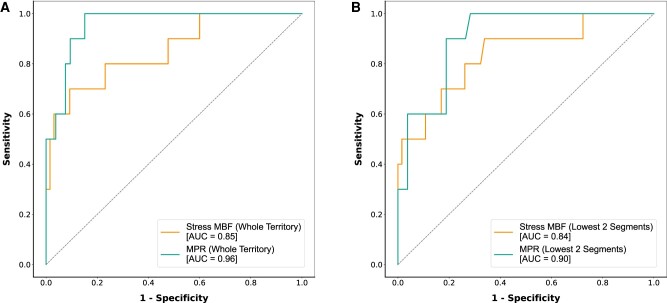
ROC curves assessing the effectiveness of quantitative perfusion map analysis in identification of functionally significant CAD (as defined by FFR ≤ 0.80) on a per-vessel level. Each curve represents a different analysis approach with both stress MBF and MPR shown. (*A*) Whole territory perfusion values. (*B*) Mean of lowest two AHA segment perfusion values.

In the patients with invasive FFR measurements, ROC analysis was performed to assess the effectiveness of territorial stress MBF and MPR in detecting an FFR value ≤0.80 on a per-territory basis. Optimal cut-off values were 1.94 mL/min/g for stress MBF and 1.97 for MPR. The ROC curves are demonstrated in *Figure 6A*. The AUC was calculated as 0.85 (0.72–0.99) for stress MBF and 0.96 (0.91–1.00) for MPR. For stress MBF: sensitivity 70.0%; specificity 90.8%; positive predictive value (PPV) 53.8%; negative predictive value (NPV) 95.2%. For MPR: sensitivity 90.0%; specificity 84.9%; PPV 52.9%; NPV 97.8%.

The same ROC analysis was also performed using the mean of the lowest two AHA segment perfusion values (*Figure 6B*). Optimal cut-off values were similar to whole territory analysis (stress MBF: 1.94 mL/min/g; MPR: 1.90), and the AUC for stress MBF was similar—0.84 (0.70–0.98), *P* = 0.646; however, the AUC for MPR was reduced—0.90 (0.82–0.99), *P* = 0.048. By using this method, sensitivity was higher, but specificity was markedly reduced (stress MBF: sensitivity 90.0%, specificity 66.2%, PPV 29.0%, NPV 97.7%; MPR: sensitivity 100.0%, specificity 71.7%, PPV 40.0%, NPV 100.0%).

### Quantitative measurements in patients with CTO and peri-infarction ischaemia

**Figure 7:**
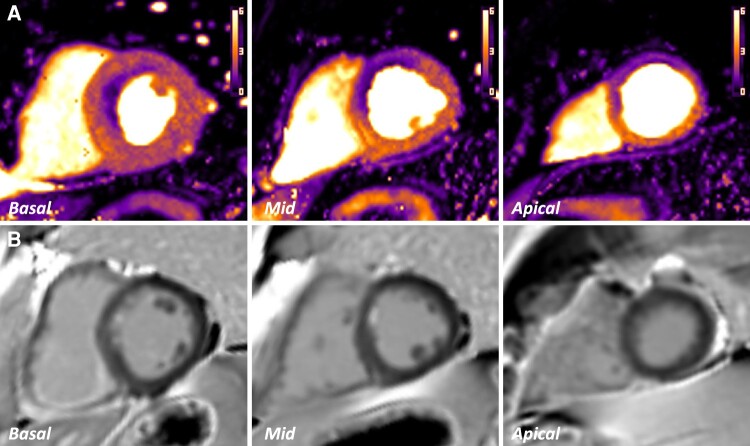
A patient with CTO and inducible perfusion defect included in analysis. (*A*) Stress MBF maps with a defect seen in the LAD artery territory (mean MBF 1.70 mL/min/g vs. 2.35 mL/min/g in the other territories). (*B*) Dark-blood LGE imaging demonstrating sub-endocardial infarction in the LAD territory, smaller in distribution than the perfusion defect seen in (*A*). ICA demonstrated a CTO in the LAD with collaterals from the circumflex artery.

Twenty patients had one or more inducible perfusion defects identified in a coronary territory which on invasive angiography appeared to be chronically occluded, with 28 individual territories used for analysis. Only mean whole territory perfusion values were analysed due to the higher prevalence of LGE scarring seen within this cohort. An example case is demonstrated in *Figure 7*.

**Figure 8:**
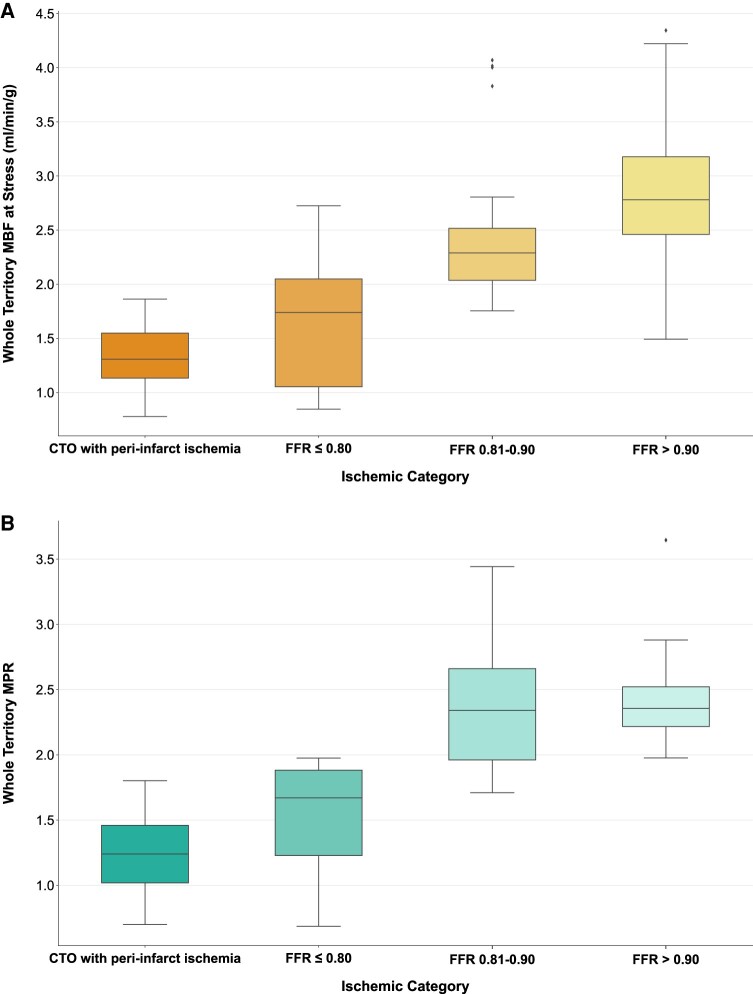
Perfusion values across ischaemic categories (defined by invasive coronary assessment). Both stress MBF (*A*) and MPR (*B*) values were significantly lower in FFR-positive and CTO categories compared with FFR-negative categories. Whole group analysis showed increased perfusion values across ischaemic categories (*P* < 0.001). No significant difference was seen between both FFR-negative categories (both stress MBF and MPR).

The median stress MBF in patients with CTO and inducible ischaemia was 1.31 (1.11–1.56) mL/min/g; rest MBF was 1.02 (0.85–1.27) mL/min/g; and MPR was 1.24 (1.01–1.46). When analysed alongside the FFR patient cohort, there was a clear trend in stress MBF and MPR values across all ischaemic categories (CTO with inducible defect, FFR ≤ 0.80, FFR 0.81–0.90, FFR > 0.90; *P* < 0.001 for both stress MBF and MPR) (*Figure 8*). When considering group-wise comparison, both stress MBF and MPR were significantly lower in CTO territories compared with both FFR-negative groups (all *P* < 0.001), but no significant difference was seen compared with the FFR-positive group (stress MBF, *P* = 0.140; MPR, *P* = 0.259).

## Discussion

This study demonstrates that the presented high-resolution free-breathing perfusion sequence with in-line automated quantitative mapping can be used to accurately detect functionally significant epicardial CAD (as defined as FFR ≤ 0.80). These findings are comparable to other automated SP-CMR quantitative mapping techniques.^20,34^ The proposed framework uses the Fermi deconvolution method which has been well validated against quantitative cardiac positron-emission tomography, invasive FFR and microsphere evaluation.^17,35,36^ Furthermore, this is the first time that automated quantitative perfusion mapping has been combined with high-resolution perfusion imaging and respiratory motion compensation, all implemented within the scanner software framework and executed on the scanner console. With high-resolution fully quantitative perfusion maps produced within 2–3  min, this technique demonstrates the potential for widespread adoption and integration of quantitative perfusion mapping in the diagnosis and risk stratification of CAD.

### Detection of functionally significant CAD

The MBF values generated from the automated process in this study, both at stress and rest, were comparable to those observed in previous studies.^14,16,17^ Given the correlation seen between FFR measurements/ischaemic categories and both stress MBF and MPR values, it may be possible to identify those patients with more severe ischaemia who could benefit from optimized medical therapy or earlier coronary intervention. Additionally, diagnostic performance of stress MBF and MPR is equivalent to other quantitative SP-CMR methods.^14,20,34^

Analysis using both ‘whole territory’ and the ‘lowest two segment’’ methods allowed a robust comparison with previous studies. As expected, the sensitivity of using the lowest two-segment analysis in detecting functionally significant CAD was higher than whole territory analysis, whilst specificity and PPV were noticeably lower. ROC analysis demonstrated similar cut-off values and performance of the two methods for stress MBF. Despite this, the AUC for MPR was lower for the lowest two-segment method, suggesting a better performance of whole territory MPR analysis.

The addition of the patients with CTO and associated inducible perfusion defect helped to emphasize a clear trend in perfusion values across ischaemic categories, with perfusion values (both stress MBF and MPR) significantly reduced in those with presumed severe ischaemia. However, with no invasive FFR measurement in these patients possible, these patients could not be included the ROC analysis for diagnostic accuracy.

With recent large clinical trials highlighting the importance of optimal medical management in many patients with CAD, non-invasive functional imaging is important in deciding the best management strategy for patients.^37,38^ With FFR-guided revascularization known to improve long-term outcomes, the accurate identification of functionally significant lesions from fully automated quantitative SP-CMR would allow clinicians to make individual management decisions from non-invasive imaging.^39^ With wider availability and good diagnostic accuracy, there is the potential for quantitative SP-CMR to augment both the diagnosis and management of myocardial ischaemia in CAD, without the need for direct visualization of the coronary arteries.

### High-resolution quantitative perfusion maps

The diagnostic accuracy of both stress MBF and MPR in predicting functionally significant CAD in this study may, in part, be due to the high-spatial resolution of the perfusion sequence and its derived quantitative mapping. Higher spatial resolution is likely to be advantageous in visual interpretation of images and perfusion maps with clearer identification of perfusion defects and a decreased impact of dark-rim artefact within the myocardial images.^10^ This study did not assess the specific advantages of high-resolution quantitative perfusion mapping—although the improved spatial resolution may allow more accurate assessment of transmural perfusion gradients, particularly in the context of myocardial infarction, where judging peri-infarction ischaemia can be difficult.

### The continuing importance of rest perfusion imaging and MPR

In this study, MPR appeared to perform better than stress MBF with regard to the sensitivity of detecting FFR-positive CAD. In a small number of patients, though, rest imaging was not acquired, most commonly due to poor baseline renal function. Recent protocol guidelines have suggested a diminished usefulness of rest imaging in diagnosing CAD and that rest perfusion sequences could be omitted from standard SP-CMR protocols.^40^ However, multiple factors may have an effect on MBF at both rest and during hyperaemia, including sex, left ventricular systolic function, and cardiac rhythm.^41,42^ Without a unifying method for correcting stress MBF for these factors, and with the good sensitivity and specificity demonstrated in this study, MPR may represent a more robust assessment of myocardial perfusion in patients with MBF values outside of typical ranges.

### Limitations of this study

As an observational study, there was no control over the number of patients referred for ICA and pressure wire-guided FFR measurements—management decisions were independently made by patients’ clinical care teams. With growing understanding of the findings from the CE-MARC2 and MR-INFORM trials in predicting the outcome and risk of CAD, fewer patients received FFR assessment during ICA than anticipated prior to revascularization.^6,9^

With a smaller number of patients with FFR measurements ≤0.80 included in the analysis compared with FFR-negative vessels, the calculated sensitivity of automated stress MBF in predicting FFR-positive lesions may be underestimated. The inclusion of the patients from the CTO subgroup would likely have increased the calculated sensitivity—though this would have assumed that all vessels within this subgroup were FFR-positive, without a valid invasive comparison. Instead, the CTO subgroup analysis was included to help balance the study cohort by demonstrating the use of the sequence in those patients with severe ischaemia. Severe ischaemia was not well represented in the FFR subgroup, with the majority of FFR-positive measurements between 0.70 and 0.80.

The analysis in this study was based on the standard coronary territories from the AHA 17-segment model and was not altered based on findings from a patient’s ICA.^33^ This ensured standardization across all patients in a bid to reduce bias. However, the stress MBF and MPR measurements within some coronary territories may be underestimated, particularly if perfusion defects straddled multiple AHA coronary territories.

Finally, the use of rate-pressure product (RPP) to correct rest MBF and MPR values was not included in the analysis. In theory, this could result in the under-estimation of perfusion reserve in those with hypertension. However, neither resting systolic blood pressure nor RPP correlated with rest MBF values in this cohort.

## Conclusions

This novel high-resolution free-breathing perfusion sequence with automated in-line quantitative mapping can accurately detect and rule out the presence of CAD. With SP-CMR being increasingly used in the diagnosis and risk stratification of patients, adoption of this technique provides a robust assessment of MBF and perfusion reserve.

## Data Availability

The data underlying this article will be shared upon reasonable request to the corresponding author.
